# High-risk motor vehicle drivers engage in more risk behaviors than motorcyclists

**DOI:** 10.1051/sicotj/2021027

**Published:** 2021-04-30

**Authors:** Kelsey A. Rankin, Theodore Zaki, Derek Ou, Chang-Yeon Kim, Leon Averbukh, Julianna R. Maisano, Michael P. Leslie, Daniel H. Wiznia

**Affiliations:** 1 Department of Orthopedics and Rehabilitation, Yale University School of Medicine New Haven CT 06510 USA

**Keywords:** Motorcycle trauma, Risky riding behavior, Motor vehicle trauma, Risk perception, Motorcycle Rider Behavior Questionnaire

## Abstract

*Objective:* The purpose of this study was to characterize and compare risk behaviors between motorcyclists and motor vehicle drivers who were involved in accidents and required hospitalization. The study focused on patients who were recently involved in motorcycle collisions (MCCs) and motor vehicle collisions (MVCs). *Methods:* We identified 63 patients involved in MCCs and 39 patients involved in MVCs who were admitted to our level-1 trauma center from April 2014 to September 2015. These 102 patients completed a questionnaire designed to evaluate risky driving behaviors. Pearson’s chi-squared tests and unpaired two-tailed *t*-tests were used to evaluate categorical and normally distributed continuous variables, respectively. Multivariable linear regression was used to analyze predictors of risk behavior. Significance was set at *p* < 0.05. *Results:* When compared to patients involved in an MCC, patients involved in MVCs were more likely to be female (*p* = 0.007), drive more frequently (*p* < 0.001), and never perceive the risk of an accident (*p* = 0.036). MVC patients were more likely to have admitted to substance use on the day of the accident (*p* = 0.030), historically drive under the influence of drugs (*p* = 0.031), drive while tired (*p* < 0.001), drive while text messaging (*p* < 0.001), and speed while overtaking vehicles (*p* = 0.011). Overall, MVC patients engaged in more risk behaviors (3.3 ± 1.3 vs. 2.0 ± 1.5; *p* < 0.001) and were more likely to engage in multiple risk behaviors (*p* < 0.001). MVCs were associated with increased risk behavior, even after controlling for protective behaviors, driving history, and demographics (*p* = 0.045). *Conclusions:* Within our cohort of trauma patients at our institution, motor vehicle drivers were more likely than motorcyclists to engage in any one risk behavior and engage in a higher number of risk behaviors. In addition, motor vehicle drivers perceived their risk of a potential accident as lower than riding a motorcycle. Education initiatives should focus on motor vehicle driver safety interventions that reduce risk behaviors.

## Introduction

Fatalities from motorcycle accidents are 27 times more likely to occur than motor vehicle accidents per vehicle mile traveled, and motorcyclists are 5 times more likely to be injured [1]. Motorcycles make up just 3% of registered vehicles, but account for 14% of total traffic accident fatalities [1–3]. The fatality rate for motorcyclists, per 100 million miles traveled, is 25.67, while that for motor vehicles is only 1.10 [2]. On average, motorcyclists travel 2311 miles/year [1], while motor vehicles travel 11,789 miles/year [3]. Therefore, there exist wide disparities in morbidity and mortality, as well as miles traveled between motorcyclists and motor vehicles.

To understand why motorcycle riding is more inherently dangerous, previous studies have examined the causes of motorcycle accidents and have categorized these into three major groups: (1) accidents caused by the motorcycle, (2) accidents caused by an environmental element (e.g., road conditions), and (3) accidents caused by human errors (e.g., drinking/substance abuse, risk behaviors, and traffic violations) [4]. While many studies have explored the first two groups which have led to the development of safety features such as helmets, protective clothing, and antilock brakes [5–9], less attention has been focused on the human element of motorcycle crashes – the third group – and particularly how this element in motorcyclists compares to that of motor vehicle drivers [10, 11].

While risk behaviors have long been implicated as contributory to both motorcycle collisions (MCCs) and motor vehicle collisions (MVCs), very few studies have compared these behaviors between MCC and MVC patient populations. Horswill et al. directly compared the behaviors of motorcyclists with those of motor vehicle drivers. Using video-based simulation, the authors found that motorcyclists are more likely than motor vehicle drivers to speed, overtake vehicles, and pull into smaller gaps in traffic [10]. An additional study compared demographic differences in motorcyclists vs. motor vehicle drivers following a driving under the influence (DUI) infraction [11] (Table 1). These were the only studies we found which compared motorcyclists to motor vehicle drivers. Therefore, there exists a significant gap in examining the existing risk behaviors in these distinct populations, and how they contribute to the likelihood of accidents.

**Table 1:** Previous studies.

Study	Cohort and method	Findings
Horswill et al. [10]	106 motorcyclists and 56 motor vehicle drivers Qualitative questionnaire	Motorcyclists were found to have increased risk behaviors, including choosing faster speeds, more frequent overtaking, and entered gaps in traffic that were smaller
Kuo et al. [11]	561 first-time offenders of driving under the influence (DUI) at mandatory educational program Anonymous self-administered questionnaire	2/3 of this cohort were motorcyclists Motor vehicle drivers reported higher rates of frequent DUI

To investigate how driver behavior influences road safety, Reason *et al.* developed the Driver Behavior Questionnaire (DBQ), which divided the human element of motor vehicle accidents into two broad categories: traffic errors (such as control operator errors) and violations (such as speeding, stunts, and failure to use safety equipment) [12]. To adapt the DBQ, an instrument developed for motor vehicle drivers, Elliott et al. revised the DBQ and developed the Motorcycle Rider Behavior Questionnaire (MRBQ) to explore the human element of motorcycle accidents, identifying five factors that were significant predictors of crash risk: traffic errors, control errors, speed violations, performance of stunts, and use of safety equipment [4]. Other studies implicate risk behaviors such as speeding, performing dangerous maneuvers or tricks, DUI, driving after drugs, texting while driving, driving while tired, switching lanes without signaling, failing to comply with road signs, and overtaking speeding vehicles as contributing factors associated with an increased risk of motorcycle collisions [13–15].

Therefore, given the limited research comparing risk behavior between motorcycle and motor vehicle drivers – our literature review revealed limited published studies on the topic – the purpose of this study was to characterize and compare human risk behaviors, as well as beliefs about perceived risk, between motorcyclists and motor vehicle drivers which contributed to collisions among hospitalized patients who were involved in a vehicular accident. We hypothesize that motorcyclists take more risks than motor vehicle drivers, and driver education should be more focused on changing behaviors in motorcyclists.

## Methods

### Materials

The study was submitted and approved by our Institutional Review Board (IRB) Office. The potential study population included all hospitalized conscious motorcycle and motor vehicle patients from April 2014 to September 2015. Any patient between the ages of 18 and 75 who experienced a motorcycle or motor vehicle accident and was consulted on by the Trauma Service in the hospital was included in the study. Qualified patients were approached by one of the investigators who explained study protocols and study goals of examining risk-taking behavior, and the subjects’ consent was obtained. Sixty-three patients who were involved in an MCC and 39 in an MVC met our selection criteria, leading to a total of 102 patients. Patient ages ranged from 18 to 68. All patients were followed up for a minimum of three months subsequent to their collision.

### Questionnaire deployment

The questionnaire was devised using Elliott et al.’s MRBQ as a starting point and incorporating questions further exploring risk behaviors implicated in studies by Lin and Kraus [13] and Tunnicliff et al. [14]. In addition, we incorporated questions to examine how patients justified their risk-taking behaviors (see Appendix). We specifically examined MCC and MVC patients who had just been admitted to the hospital to survey their immediate reflections of their risk behavior. The questionnaire included *demographic information, questions to evaluate whether patients have habits of risk behaviors, activities at the time of the accident, driving history and perceived skill level, and patients’ understanding of the operative treatment plan* (see Appendix).

Draft questionnaires were tested by a small group of trauma patients. Patients were asked to highlight questions that were difficult to understand or answer, and revisions were made based on their feedback. Clinical data were obtained from the electronic medical record as well as our institution’s contribution to the National Trauma Databank (NTDB).

### Variables

Collected demographic variables included gender (male/female), age (integer, treated as continuous), race/ethnicity (White, Hispanic, Black, Asian, other), marital status (single, married, divorced, separated, widowed, relationship, other), highest level of education (high school/GED, college, trade school, advanced graduate work, other), and occupation (student, part-time, full-time, retired, unemployed).

Driving history variables included number of previous accidents (continuous; but was transformed into a dummy binary variable of “previous accident” (Y/N)), driving experience (everyday, several times/week, once a week, a few times a month, does not drive at all, does not own a car), previously imagined possibility of accident (never, a little bit, sometimes, frequently, always, impossible to answer; this was transformed into a dummy binary variable of “never imagined being in an accident before” (Y/N)), distraction present during accident (no, does not remember, animal, other vehicle did not follow proper driving protocol, reckless driving, road/weather conditions, speeding, using the telephone/texting, other; this was transformed into a dummy binary variable “distraction present” (Y/N), as well as dummy binary variable “other vehicle did not follow proper driving protocol” (Y/N)).

Risk behavior variables included history of smoking (Y/N), pack per day (PPD) in smokers (continuous), admitted substance use (Y/N), pain medication (Y/N), or alcohol use (Y/N) at the time of the accident, frequency of drinks/week (integer; transformed into dummy binary variable “drinking ≥ 4 times/week” (Y/N)), number of drinks at a time (integer; transformed into dummy variable “drinking ≥ 5 drinks at a time” (Y/N)), speeding (Y/N), dangerous maneuvers/tricks (Y/N), DUI of alcohol (Y/N), DUI of drugs (Y/N), driving while tired (Y/N), driving and texting (Y/N), switching lanes without signaling (Y/N), failure to comply with road signs (Y/N), and speeding while overtaking vehicles (Y/N). Aggregate risk behavior score was a compound score that included: speeding, dangerous maneuvers/tricks, DUI of alcohol, DUI of drugs, driving while tired, driving and texting, switching lanes without signaling, failure to comply with road signs, and speeding while overtaking vehicles, alcohol on the day of the accident, substance use on the day of the accident, and pain medication on the day of the accident.

Clinical outcome variables included length of stay (LOS) measured in days (integer, treated as continuous), length of intensive care unit (ICU) stay (integer; also transformed into dummy binary variable “ICU stay > 0 days” (Y/N)), length of intubation (integer; also transformed into dummy binary variable “intubation > 0 days” (Y/N)), positive drug test (Y/N), blood alcohol concentration (BAC) (continuous; also transformed into dummy binary variable “BAC > 0” (Y/N)), systolic blood pressure (BP) (integer, treated as continuous), Glasgow Coma Scale (GCS) (integer, treated as continuous), head injury (read by CT; Y/N), cervical injury (read by CT; Y/N), injury severity score (ISS) (integer, treated as continuous), and hospitalization required surgery (Y/N). ISS was calculated by our institution’s clinical registry NTDB nurses.

### Statistical analysis

Categorical variables were analyzed using Pearson’s chi-squared test. Integer variables were treated as continuous. All continuous variables were tested for normality using the Shapiro–Wilk test and normally distributed variables were analyzed using unpaired, two-tailed *t*-tests. Multivariable linear regressions were used to assess predictors of engaging in risk behavior. Statistical analysis was performed using Stata v.16.1. Significance was set at *p* < 0.05.

## Results

### Collision demographics

Overall, there were no large differences in patient demographics between groups. There were no observed differences in patient age (MCC: 38.8 ± 13.2 vs. MVC: 39.3 ± 13.3; *p* = 0.870), race (*p* = 0.296), marital status (*p* = 0.798), education level (0.058), or occupation (0.299). Education level was nearly significant, with 55.6% of MCC patients having a high school/GED vs. 35.9% of MVC patients. There were significantly more women involved in MVC (30.8%) vs. MCC (9.5%) (*p* = 0.007) (Table 2).

**Table 2:** Patient demographic data.

Category	MCC (*n* = 63)	MVC (*n* = 39)	*p*-value
Age (Mean + SD)	38.8 + 13.2	39.3 + 13.3	0.870
Female (% (*n*))	9.5% (6)	30.8% (12)	**0.007**
*Race/ethnicity* (% (*n*))			
White	74.6% (47)	71.8% (28)	
Hispanic	11.1% (7)	7.7% (3)	0.296
Black	12.7% (8)	12.8% (5)	
Other/unknown	1.6% (1)	7.7% (3)	
*Marital status*			
Single	50.7% (32)	59.0% (23)	0.778
Married	33.3% (21)	28.2% (11)	
Other/unknown	15.9% (10)	12.8% (5)	
*Highest level of education*			
High school/GED	55.6% (35)	35.9% (14)	
Trade school	3.2% (2)	0.0% (0)	
College	28.6% (18)	35.9% (14)	0.058
Advanced graduate work	7.9% (5)	7.7% (3)	
Other/unknown	4.8% (3)	2.1% (8)	
*Occupation*			
Student	4.8% (3)	5.1% (2)	
Full-time:	69.8% (44)	66.7% (26)	
Construction	29.5% (13)	19.2% (5)	
Other	70.5% (31)	80.8% (21)	
Part-time:	11.1% (7)	5.1% (2)	0.299
Construction	0.0% (0)	0.0% (0)	
Other	100.0% (7)	100.0% (2)	
Retired	4.8% (3)	5.1% (2)	
Unemployed	4.8% (3)	18.0% (7)	
Other/unknown	4.8% (3)	0.0% (0)	

MCC: motorcycle collision; MVC: motor vehicle collision; SD: standard deviation; GED: general education development certificate. Significant *p*-values bolded.

### Patient driving history

Patients in an MVC drove more, but worried about accidents less. Patients involved in an MVC were more likely to drive every day (MCC: 32.8% vs. MVC: 89.7%; *p* < 0.001), and also more likely to have never imagined the possibility of an accident prior to the accident in question (MCC: 50.8% vs. MVC: 71.8%; *p* = 0.036). There were no differences in having previously been involved in an accident (*p* = 0.261) (Table 3A).

**Table 3:** Patient risk behaviors.

Category	MCC (*n* = 63)	MVC (*n* = 39)	*p*-value
A. Driving history			
Previously in accident	100.0% (61)	92.3% (36)	0.261
*Driving experience*			
Drive every day	32.8% (20)	89.7% (35)	**<0.001**
Drive several times/week	31.2% (19)	2.6% (1)	
Drive once a week	11.5% (7)	2.6% (1)	
Drive a few times a month	19.7% (12)	2.6% (1)	
Doesn’t drive at all	4.9% (3)	0.0% (0)	
Doesn’t own a car	0.0% (0)	2.6% (1)	
*Introspection of driving*			
Never imagined possibility of accident before	50.8% (32)	71.8% (28)	**0.036**
Distraction present	58.7% (37)	59.0% (23)	0.981
Other vehicle did not follow proper driving protocol	44.4% (28)	30.8% (12)	0.169
B. Substance use at time of accident			
*Smoking History*			
Smoker	37.7% (23)	38.5% (15)	0.939
PPD amongst smokers (Mean + SD)	0.7 + 0.3	0.5 + 0.3	0.159
*Drinking History*			
Drinking > 4 times/week	12.7% (8)	5.1% (2)	0.212
Drinking > 5 drinks at a time	6.4% (4)	10.3% (4)	0.476
Admitted alcohol on day of accident	19.4% (12)	23.1% (9)	0.654
*Substance Abuse at Time of Accident*			
Admitted substance use	3.3% (2)	15.4% (6)	**0.030**
Admitted pain medication	4.9% (3)	5.1% (2)	0.962
C. Risk behavior history			
Admitted speeding	71.4% (45)	79.5% (31)	0.364
Admitted dangerous maneuvers or tricks	17.5% (11)	7.7% (3)	0.164
Admitted driving under influence of alcohol	17.5% (11)	23.1% (9)	0.487
Admitted driving under influence of drugs	6.4% (4)	20.5% (8)	**0.031**
Admitted driving while tired	36.5% (23)	79.5% (31)	**<0.001**
Admitted driving and texting	12.7% (8)	48.7% (19)	**<0.001**
Admitted switching lanes without signaling	1.6% (1)	2.6% (1)	0.730
Admitted failure to comply with road signs	1.6% (1)	2.6% (1)	0.730
Admitted speeding while overtaking vehicles	38.1% (24)	64.1% (25)	**0.011**
Risk behavior aggregate score (mean + SD)	2.0 + 1.5	3.3 + 1.3	**<0.001**
>1 risk behavior	58.7% (37)	94.9% (37)	**<0.001**
			

MCC: motorcycle collision; MVC: motor vehicle collision. PPD: pack per day; SD: standard deviation. Significant *p*-values bolded.

### Patient risk behaviors

MVC patients were more likely to engage in any risk behavior, and to engage in more risk behaviors. MVC patients were more likely to have admitted to substance use at the time of the accident (MCC: 3.3% vs. MVC: 15.4%; *p* = 0.030). There were no observed differences in smoking history (0.939) or PPD use in smokers (*p* = 0.159), drinking on the day of the accident (*p* = 0.654), history of drinking frequency (*p* = 0.212) or binge drinking (*p* = 0.476), or use of pain medication on the day of the accident (0.962) (Table 3B).

MVC patients were more likely to admit to DUI of drugs (MCC: 6.4% vs. MCC: 20.5% *p* = 0.031), driving while tired (MCC: 36.5% vs. MVC: 79.5%; *p* < 0.001), driving while texting (MCC: 12.7% vs. MVC: 48.7%; *p* < 0.001), and speeding while overtaking other vehicles (MCC: 38.1% vs. MVC: 64.1%; *p* = 0.011). There were no observed differences in admitted speeding (*p* = 0.364), dangerous maneuvers/tricks (*p* = 0.164), alcohol use while driving (*p* = 0.487), switching lanes without signaling (*p* = 0.730), or failure to comply with road signs (*p* = 0.730). Overall, MVC patients had more risk behaviors (mean score MCC: 2.0 ± 1.5 vs. MVC: 3.3 ± 1.3; *p* < 0.001), and were more likely to engage in multiple risk behaviors (MCC: 58.7% vs. MVC: 94.9%; *p* < 0.001) (Table 3C).

### Patient clinical outcomes

MVC patients had longer hospitalizations, but had lower GCS scores. MVC patients had an increased LOS (MCC: 8.8 ± 8.6 vs. MVC: 12.9 ± 11.6; *p* = 0.046). MCC patients had a higher GCS score (MCC: 14.9 ± 0.5 vs. MVC: 13.7 ± 3.3; *p* = 0.007). No differences were observed between length of ICU stay (*p* = 0.845), ICU > 0 days (*p* = 0.227), intubated > 0 days (*p* = 0.286), positive drug test (*p* = 0.504), blood alcohol concentration (BAC) > 0 (*p* = 0.098), systolic blood pressure (BP) (*p* = 0.523), head injury (*p* = 0.065), cervical injury (*p* = 0.924), injury severity score (ISS) (*p* = 0.084), or requiring surgery (*p* = 0.990) (Table 4).

**Table 4:** Patient clinical data.

Category	MCC (*n* = 63)	MVC (*n* = 39)	*p*-value
Length of hospital stay (mean days + SD)	8.8 + 8.6	12.9 + 11.6	**0.046**
Length of ICU stay (mean days + SD)	0.6 + 1.2	0.6 + 0.9	0.845
% in ICU > 0 days	33.3% (20)	43.6% (17)	0.227
% intubated > 0 days	4.9% (3)	10.3% (4)	0.286
% positive drug test	42% (26)	48.7% (19)	0.504
% BAC > 0	24.2% (15)	41.0% (16)	0.098
Systolic BP (mean + SD)	126.6 + 20.3	124.1 + 17.1	0.523
GCS (mean + SD)	14.9 + 0.5	13.7 + 3.3	**0.007**
% head injury	9.7% (6)	23.1% (9)	0.065
% cervical injury	9.7% (6)	10.3% (4)	0.924
ISS (mean + SD)	11.2 + 6.9	13.8 + 7.7	0.084
% required surgery	87.1% (54)	87.2% (34)	0.990

MCC: motorcycle collision; MVC: motor vehicle collision; ICU: intensive care unit; BAC: blood alcohol concentration; BP: blood pressure; GCS: Glascow Coma Scale; ISS: injury severity score. Significant *p*-values bolded.

### Predictors of risk behavior

We next assessed significant predictors of engaging in increased risk behaviors. Univariate analyses showed that MVC patients were more likely to engage in increased risk behavior (MVC coefficient: 1.41; *p* < 0.001). Next, we controlled for all associated protective factors in both motorcycle driving and motor vehicle driving, driving history, and all demographic covariates. Here, we found that MVC patients were still more likely to engage in increased risk behaviors (coefficient: 1.86; *p* = 0.045), and in fact having been in an MVC crash was the only variable that could predict increased risk behavior (Table 5).

**Table 5:** MVC associated with increased risk behavior after controlling for demographic and protective factors.

Crash type	Total risk behavior (coefficient)	*p*-value
MVC	1.86	**0.045**
*Demographic factors*		
Female	−0.68	0.256
*Race/ethnicity*		
Asian	−0.84	0.643
Black	0.06	0.918
Hispanic	0.11	0.880
White	Referent	
Other/unknown	0.08	0.955
Age	−0.03	0.206
*Marital status*		
Single	Referent	
Married	−0.88	0.060
Divorced	−0.44	0.569
Other	−0.99	0.384
*Education*		
High school/GED	Referent	
College	0.25	0.558
Graduate	0.46	0.510
Trade school	1.35	0.289
Other/unknown	0.33	0.673
*Occupation*		
Part-time	−0.33	0.623
Full-time	Referent	
Student	−0.37	0.687
Unemployed	−1.09	0.101
Retired	0.29	0.745
*Protective factors*		
Helmet	0.05	0.926
Seatbelt	−0.09	0.906
*Length of time driving*		
A few months	Referent	
Less than a year	−3.94	0.064
1–3 years	−0.97	0.480
3–10 years	−0.87	0.538
> 10 years	−0.51	0.716
*Driving frequency*		
Not at all	Referent	
Doesn’t own a car	−1.32	0.543
A few times a month	−0.33	0.789
Once a week	−0.64	0.789
Several times a week	−0.42	0.725
Everyday	−0.20	0.864
*Driving skill*		
Beginner	−0.75	0.612
Novice	−2.09	0.177
Intermediate	0.44	0.544
Advanced	0.14	0.811
Professional	Referent	
Accident before	0.67	0.604

MVC: motor vehicle collision; GED: general educational development certificate. Significant *p*-values bolded.

## Discussion

We sought to characterize and compare risk behaviors in MCC and MVC patient populations, and how they may contribute to the likelihood of collisions. Overall, we found that motor vehicle drivers are more likely to engage in risk behaviors, and engage in multiple risk behaviors. They also perceived a lower risk of being in an accident, compared to motorcyclists.

There are several limitations to this study. Demographically, study participants were predominantly older, male drivers in New England, USA. As such, risk behaviors among drivers younger than 25 who are at higher risk of injury and death following both MCC and MVCs were not well represented. In addition, because the study was based on self-reported data, our conclusions are subject to recall bias. Additionally, despite the anonymity of the questionnaire, participants may have been unwilling to disclose their illegal behaviors or may have provided answers they considered more desirable to investigators.

An important limitation to note is the discrepancy of driving exposure: motor vehicle drivers have significantly more road time than motorcyclists. This increases the likelihood of an accident. Another limitation of this study is its small sample size of 102 patients. In addition, this study may also suffer from a collection bias, because patients with severe injuries may have died prior to being admitted to the hospital, as well as the notion that we are only examining risk behaviors in a cohort of patients who had an accident, and we, therefore, cannot apply our findings to the overall driving population.

We found that overall, MVC patients engaged in more risk behaviors. They were also more likely to engage in multiple risk behaviors. This remained true even when controlling for protective behaviors, driving history, and demographic factors. This is in contrast to previous findings, which demonstrated that motorcyclists were more likely to engage in risk behaviors [10]. These differences may be the result of regional differences in behavior, as the previous study was conducted in the United Kingdom, and this study was conducted in New England, USA. These conflicting results suggest a need for further investigation into the differing risk behaviors to better provide educational and legislative advice specific to motorcyclists and motor vehicle drivers.

We found that while MVC patients were more frequent drivers, they were less likely to perceive the risk of an accident. This suggests an increased level of comfort and safety as compared with the MCC cohort. The perceived decreased risk of an accident is demonstrated in our findings, as MVC patients were more likely to report more risk behaviors than MCC patients. This could reflect a false sense of security and confidence and possibly be an example of risk compensation due to their increased sense of safety.

Motorcyclists are likely more cautious drivers for several reasons: most importantly the increased vulnerability of riding outside a vehicle, less protection present compared with a motor vehicle, increased likelihood of mortality during a crash [1–3], as well as the high level of coordination, attention, and motor skills that are required to safely drive a motorcycle [16]. Additionally, several risk behaviors we looked at would be challenging for motorcyclists, such as texting while driving. For example, it is more difficult to recover from a control error when riding a motorcycle compared to driving a motor vehicle [4]. Thus, while Horswill et al. found that motorcyclists are riskier than motor vehicle drivers on video-based assessments [10], our study suggests that in actuality, motorcyclists report engaging in fewer risk behaviors and are more concerned and cautious about accidents than motor vehicle drivers.

In terms of clinical outcomes following MVC vs. MCC accidents, motorcyclists had a higher GCS, but a shorter LOS. Otherwise, there were no significant differences in clinical outcomes. It has been demonstrated that motorcyclists experience increased risk of fatality and severe injury compared to motor vehicle drivers [17–20].

Demographically, our patient populations in both cohorts were similar with respect to age, race, marital status, level of education, and occupation. However, a significantly increased proportion of males was noted in the MCC cohort. This finding is consistent with multiple studies that have reported that men comprise a larger proportion of MCC patients [21–24].

It is interesting to note that there were no differences between the two groups in the presence of a distraction during the accident. For example, participants did not report differences in whether another vehicle caused the accident by violating safe driving practices. This is in contrast to previous literature, which has demonstrated that unlawful driving practices of other vehicles are a major cause of motorcycle accidents [1].

In conclusion, motor vehicle drivers who were involved in accidents were more likely than motorcyclists to admit to engaging in risk behaviors such as driving after abusing drugs, speeding while overtaking other vehicles, and driving while tired. Overall, they also engaged in more risk behaviors. Additionally, our results suggest that motor vehicle drivers were less concerned about the potential risk of an accident than motorcyclists do, suggesting that motor vehicle drivers may not fully appreciate the risks of driving. This decreased level of caution may contribute to their increased likelihood of engaging in risk behaviors. These findings underscore the importance of road safety interventions in high-risk drivers which focus on reducing behavioral violations, particularly among motor vehicle drivers.

## Conflicts of interest

The authors report no conflicts of interest.

## Appendix

### Motorcycle and Motor Vehicle Registry Questionnaire

Demographic data

- Age:
- Sex: Male/Female
- What is your marital status?
  - Single
  - Married
  - Separated/widowed/divorced
- What is your highest level of education?
  - High school/GED
  - College
  - Bachelor’s Degree
  - Master’s Degree
  - Advanced graduate work or PhD
  - Not sure
- What is your occupation?
  - Student
  - Full-time work construction
  - Full-time work non-construction
  - Part-time work construction
  - Part-time work non-construction
  - Unemployed
  - Retired

Smoking history

- Do you smoke (y/n)
  - If yes, how many PPD? _________

Drinking history

- How often did you have a drink containing alcohol in the past year?
  - Never
  - Monthly or less
  - Two to four times a month
  - Two to three times a week
  - Four or more times a week
- How many drinks did you have on a typical day when you were drinking in the past year?
  - None, I do not drink
  - 1 or 2
  - 3 or 4
  - 5 or 6
  - 7 to 9
  - 10 or more
- Did you have any alcoholic beverages on the day of the accident?
  - yes/no

Substance abuse at time of accident

- Were you using any substances just prior to the accident?
  - yes/no
- If yes, which drugs?
  - Marijuana
  - Crack
  - Cocaine
  - Heroine
  - PCP
  - Other____________
- Were you taking any pain medication at the time of the accident, such as oxycodone, methadone, Percocet, Vicodin, Dilaudid, etc.?
  - yes/no

Introspection of health

- How well do you understand your injuries?
  - Poor understanding, I am not sure what is wrong
  - Adequate understanding, I understand what is wrong, but not all the details
  - Good understanding, I know exactly what is wrong
- How well do you understand the potential complications, such as not being able to use your injured limb, possible amputation, etc.?
  - Poor understanding, I do not understand the potential complications
  - Adequate understanding, I understand the general complications
  - Good understanding, I know the exact complications that could happen
- How well do you understand the treatment plan?
  - Poor understanding, I am not sure what the plan is
  - Adequate understanding, I understand the plan, but not all the details
  - Good understanding, I know the plan exactly

Driving behavior

- How many motorcycle/motor vehicle accidents have you been involved in?
  - 1, 2, 3, 4, 5, or more
- How long have you driven a motorcycle/motor vehicle?
  - Less than a year
  - 1–3 years
  - 3–10 years
  - >10 years(5)
- How frequently did you drive before the accident?
  - Not at all
  - A few times a month
  - Once a week
  - Several times a week
  - Daily
- How would you describe your driving skill?
  - Novice
  - Beginner
  - Intermediate
  - Advanced
  - Professional

Introspection of driving

**Table:**

	Never (0% of the time)	A little bit (1–25% of the time)	Sometimes (26–50% of the time)	Frequently (51–75% of the time)	Always (76–100% of the time)
Before your latest accident, how often did you think you would have an accident?					

- Were there any distractions that may have caused the accident such as (*circle all that apply*)
  - Using the telephone, texting
  - Reckless driving
  - Speeding
  - Other vehicle did not follow proper driving protocol
  - Road or weather conditions
  - None

Risk behaviors

**Table:**

	Never (0% of the time)	A little bit (1–25% of the time)	Sometimes (26–50% of the time)	Frequently (51–75% of the time)	Always (76–100% of the time)
How frequently would you ride above the speed limit?					
How frequently would you perform dangerous maneuvers or tricks?					
How frequently do you drive under the influence of alcohol?					
How frequently do you drive under the influence of other drugs?					
How frequently do you drive while texting or talking on a cell phone?					

Traffic violations

**Table:**

	Never (0% of the time)	A little bit (1–25% of the time)	Sometimes (26–50% of the time)	Frequently (51–75% of the time)	Always (76–100% of the time)
How often did you ride while feeling tired?					
Do you signal before switching lanes?					
Do you obey all road signs (stop[author’s]lights, stop signs, yield signs, etc.)?					
When you overtake vehicles, are they traveling above the speed limit?					
